# ERK/Drp1-dependent mitochondrial fission is involved in the MSC-induced drug resistance of T-cell acute lymphoblastic leukemia cells

**DOI:** 10.1038/cddis.2016.370

**Published:** 2016-11-10

**Authors:** Jianye Cai, Jiancheng Wang, Yinong Huang, Haoxiang Wu, Ting Xia, Jiaqi Xiao, Xiaoyong Chen, Hongyu Li, Yuan Qiu, Yingnan Wang, Tao Wang, Huimin Xia, Qi Zhang, Andy Peng Xiang

**Affiliations:** 1 Program of Stem Cells and Regenerative Medicine, Affiliated Guangzhou Women and Children's Hospital, Zhongshan School of Medicine, Sun Yat-Sen University, Guangzhou, China; 2 Center for Stem Cell Biology and Tissue Engineering, Key Laboratory for Stem Cells and Tissue Engineering, Ministry of Education, Sun Yat-Sen University, Guangzhou, China; 3 Biotherapy Center, The Third Affiliated Hospital, Sun Yat-Sen University, Guangzhou, China; 4 Key Laboratory of Industrial Fermentation Microbiology, Ministry of Education, College of Biotechnology, Tianjin University of Science and Technology, Tianjin, China; 5 Department of Pediatrics, Sun Yat-Sen Memorial Hospital, Sun Yat-Sen University, Guangzhou, China; 6 Department of Biochemistry, Zhongshan School of Medicine, Sun Yat-Sen University, Guangzhou, China

## Abstract

The bone marrow microenvironment facilitates the proliferation and survival of leukemia cells, contributing to disease relapse. Bone marrow-derived mesenchymal stem cells (MSCs) are well known to promote cancer chemoresistance via soluble factors and cell adhesion. However, little is known about the effects of MSCs on the mitochondrial dynamics of T-cell acute lymphoblastic leukemia (T-ALL) cells, or how this may influence the chemoresistance of these cells. Here, we tested both indirect (Transwell) and direct coculture strategies, and found that MSCs protected T-ALL cells from chemotherapeutic cell death and cytotoxicity under both culture conditions. In addition, cell viability was higher in the direct contact system compared with the Transwell system. We further showed that exposure of T-ALL cells to MSCs decreased mitochondrial reactive oxygen species (ROS) levels and promoted a pro-glycolytic shift that was characterized by increased glucose uptake and lactate production with concomitant reductions in adenosine triphosphate production and mitochondrial membrane potential. In T-ALL cells cocultured with MSCs, the mitochondrial morphology of T-ALL cells were altered from elongation to fragmentation because of the extracellular signal-regulated kinase activation-mediated phosphorylation of the pro-fission factor, dynamin-related protein 1 (Drp1), at residue S616. Consistent with this, the expression of S616-phosphorylated Drp1 recapitulated the mitochondrial dynamics, mitochondrial ROS levels, metabolic switching and chemoresistance seen in T-ALL cells cocultured with MSCs. These findings suggest that the ability of MSCs to trigger Drp1 activation-induced changes in mitochondrial dynamics is crucial to their ability to protect cells against chemotherapeutic agents.

T-cell acute lymphoblastic leukemia (T-ALL) is one of the most aggressive hematologic malignancies. It arises from the malignant transformation of T-cell progenitors and accounts for 10–15% pediatric and 25% adult ALL cases.^1^ Clinically, T-ALL is treated with the high-dose multi-agent chemotherapy, which has improved the cure rate to over 75% in children and about 50% in adults.^2^ Nevertheless, many T-ALL patients experience primary chemoresistance and leukemia relapse because of minimal residual disease (MRD). These issues remain major challenge in our efforts to cure T-ALL.^3, 4^

An increasing number of studies suggest that the bone marrow microenvironment, especially the mesenchymal stem cells (MSCs) in bone marrow, may promote drug resistance and protect leukemia cells from apoptosis. It is widely known as the environment-mediated drug resistance (EMDR).^5, 6^ Two drug resistance forms generally participate in MSC-mediated leukemia cell survival and chemoresistance: soluble factor-mediated drug resistance (SFM-DR), which reflects indirect communications through MSC-secreted cytokines, chemokines and growth factors; and cell adhesion-mediated drug resistance (CAM-DR), which is induced by the direct contact of MSCs and leukemia cells mainly through integrin family proteins and the extracellular matrix.^7, 8^ Many preclinical studies have verified that therapies targeting EMDR pathways can increase the efficacy of chemotherapy.

A large body of work has investigated the potential mechanisms of chemotherapy. Many different signaling pathways have been reported participated in chemoprotection after the interactions between leukemia cells and stromal cells. Krampera *et al.* have demonstrated the anti-apoptotic role of Notch signaling in MSC-induced leukemia cells survival.^9, 10, 11^ In addition, the induction of intracellular oxidative stress, which has been shown to be an important anticancer mechanism of chemotherapeutic agents, can result in the preferential killing of leukemia cells.^12, 13^ Given that mitochondria are the key source for reactive oxygen species (ROS), it seems logical that targeting the respiratory chain and increasing mitochondrial ROS levels in leukemia cells could promote cytotoxicity. For example, Jitschin *et al.*^14^ reported that treatment of chronic lymphocytic leukemia (CLL) cells with PK11195, which blocks the mitochondrial F1F0-ATPase, increased the generation of mitochondrial ROS and induced cell death. It is widely accepted that mitochondria are central sites of bioenergetics in all cells, including cancer cells.^15^ Cancer cells exhibit a metabolic phenotype distinct from that of normal cells, which is called aerobic glycolysis or the Warburg effect. This change in metabolism is characterized by the glucose uptake and the excess lactate production in the presence of oxygen.^16, 17^ However, we know little about the effects of MSCs on mitochondrial dynamics in T-ALL cells, or how these effects may contribute to the ability of MSCs to protect leukemia cells against chemotherapies.

Here, we showed that coculture with MSCs enabled T-ALL cells to resist chemotherapeutic cytotoxicity, and reported for the first time that this was associated with mitochondrial ROS levels decrease, pro-glycolytic phenotype switching and mitochondrial fragmentation, all of which were triggered by extracellular signal-regulated kinase (ERK) activation-induced dynamin-related protein 1 (Drp1) phosphorylation. Taken together, our findings showed that mitochondrial dynamics and redox characteristics could be candidate therapeutic targets for new strategies aimed at T-ALL treatment.

## Results

### MSCs inhibit the chemotherapeutic cell death and cytotoxicity of T-ALL cells *in vitro*

To establish appropriate concentrations of cytosine arabinoside (Ara-C) and methotrexate (MTX) for inducing cytotoxicity in T-ALL cells (Jurkat and primary T-ALL cells), we used the cell counting kit-8 (CCK-8) assay to examine cells treated with increasing drug concentrations for 48 h. The results showed that Ara-C and MTX significantly and dose dependently reduced the viability of T-ALL cells (Supplementary Figure 1A), with IC50 values of ~271.3 and 65.9 nM for Jurkat and ~5.32 and 1.23 *μ*M for primary T-ALL cells, respectively. Next, we used 300 nM Ara-C and 100 nM MTX for Jurkat and 6 *μ*M Ara-C and 1.5 *μ*M MTX for primary T-ALL cells, and detected the viability of cells at different time points. Our results showed that the viabilities of Ara-C- and MTX-treated T-ALL cells decreased time dependently (Supplementary Figure 1B).

**Figure 1:**
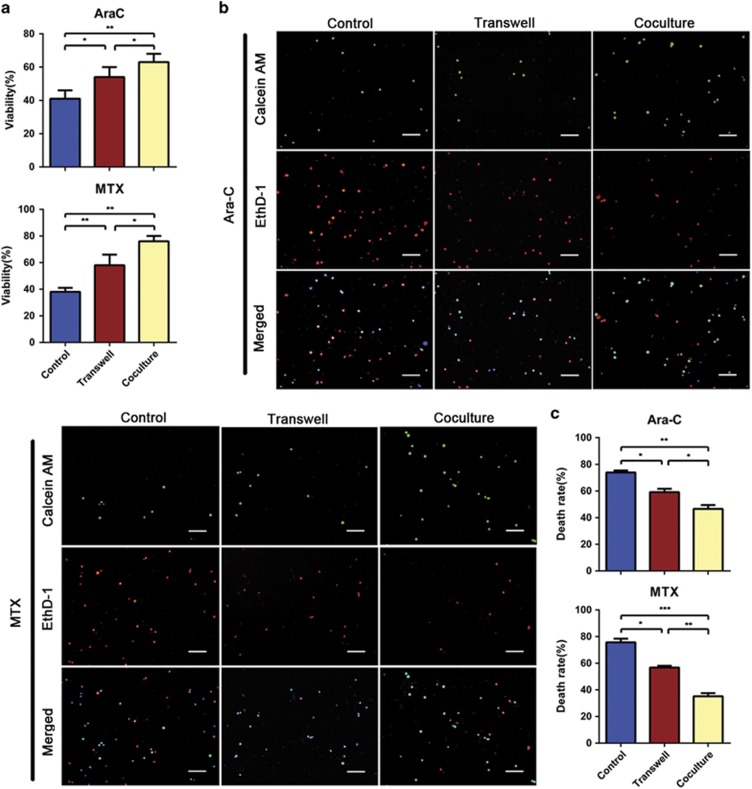
MSCs protect T-ALL cells from chemotherapeutic cell death and cytotoxicity *in vitro*. (**a**) Cell viability was assessed with the CCK-8 assay. Data are presented as the mean±S.E.M. (*n*=3) for each group. (**b**) Apoptosis of Jurkat cells was also determined by probing with calcein AM/EthD-1. Scale bar, 50 *μ*m. (**c**) The percentage of dead cells (EthD-1-positive) was calculated. Results are presented as the mean±S.E.M. from at least 500 cells counted (*n*=3) (*P<0.05; **P<0.01; ***P<0.001; *t*-test)

Then, we exposed the T-ALL cells to chemotherapeutic drugs in Transwell and coculture systems with MSCs, and examined the MSCs characteristics and Jurkat cells viability. As shown in Supplementary Figure 2, MSCs still maintained the properties of multipotent stem cells when cocultured with Jurkat cells with or without chemotherapeutic agents. In addition, MSCs protected Jurkat cells from Ara-C/MTX-induced cytotoxicity in both Transwell and direct coculture systems. To further confirm these protective effects, the Jurkat cell death rates in the presence of Ara-C or MTX were measured using LIVE/DEAD viability/cytotoxicity assays. The results showed that MSCs promoted Jurkat cell survival more remarkably through direct contact (Figures 1b and c). We also found that the apoptosis levels of primary T-ALL cells were significantly decreased by direct contact coculture (Supplementary Figure 3). These results indicate that MSCs protect T-ALL cells from chemotherapeutic cytotoxicity *in vitro*, which seems to involve both direct cell–cell contact and MSC-secreted soluble factors.

### The MSC-induced chemoresistance of T-ALL cells is due to reduced mitochondrial ROS levels

**Figure 2:**
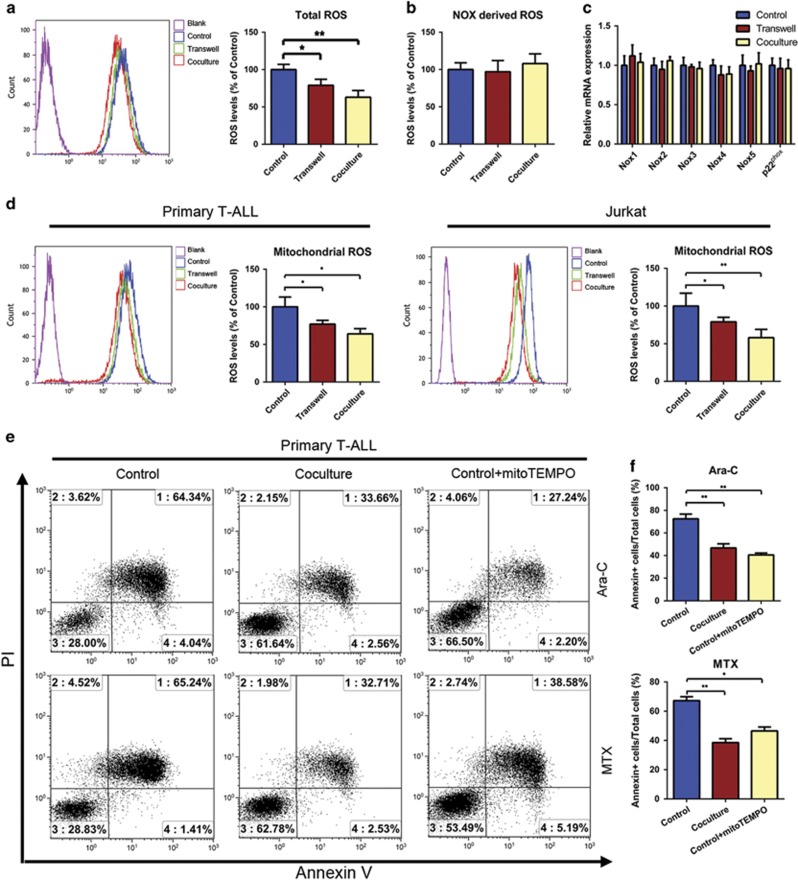
The increased chemoresistance of MSC-cocultured T-ALL cells is due to reduced mitochondrial ROS levels. (**a**) ROS generation was significantly decreased in primary T-ALL cells subjected to direct or indirect coculture with MSCs compared with mono-cultured. (**b**) Total superoxide production in primary T-ALL cells cultured with or without MSCs was measured using the Diogenes probe. (**c**) The Nox gene expression levels of primary T-ALL cells cultured with or without MSCs were examined by qRT-PCR. (**d**) MSCs coculture significantly decreased the generation of mitochondrial ROS in T-ALL cells. (**e**) Cells were treated with MitoTEMPO and the chemotherapeutic agents, and apoptosis was measured by Annexin V/PI staining and FACS. (**f**) The percentage of apoptotic cells in each group was analyzed and graphed. Results above are expressed as the mean±S.E.M. of three independent experiments (*P<0.05; **P<0.01; *t*-test)

Accumulating evidence has demonstrated that ROS and cellular oxidative stress are responsible for more than just tumor initiation and malignancy,^18^ and the excessive ROS induced by chemotherapeutic agents can be toxic to tumor cells.^19^ Our flow cytometric analysis of the intracellular redox status showed that primary leukemia cells cocultured with MSCs exhibited less intracellular ROS generation than those mono-cultured in suspension (Figure 2a). The major sites of cellular ROS generation include the mitochondrial electron transport chain^20^ and the NADPH oxidase (Nox) complex.^21^ However, we failed to find any significant difference in the extracellular superoxide generation of primary leukemia cells cultured with and without MSCs using the Diogenes probe (Figure 2b). Moreover, quantitative real-time polymerase chain reaction (qRT-PCR) showed that the gene expression levels of Nox were not altered in primary leukemia cells cultured in direct contact with MSCs (Figure 2c). Similar results were also detected in Jurkat cells (Supplementary Figures 4A-4C). However, when we detected the mitochondrial ROS levels using MitoSOX, we found that both primary T-ALL cells and Jurkat cells in direct contact with MSCs exhibited significantly lower mitochondrial ROS levels (Figure 2d). These results show that a change in the mitochondrial ROS levels contributes greatly to the decreased intracellular ROS observed in T-ALL cells cocultured with MSCs.

To confirm the decrease in mitochondrial ROS levels is important to the chemoresistance of T-ALL cells, we treated mono-cultured cells with MitoTEMPO (50 *μ*M; a specific mitochondrial ROS scavenger) for 6 h and observed the decreased mitochondrial ROS levels (Supplementary Figure 4D) and reduced percentage of Annexin V-positive cells (Figures 2e and f) in the presence of chemotherapeutic drugs. Taken together, our results reveal the MSC-mediated chemoprotection of T-ALL cells depends on the mitochondrial ROS levels regulation.

### Mitochondrial metabolism switches toward the glycolytic phenotype in T-ALL cells cultured directly or indirectly with MSCs

**Figure 3:**
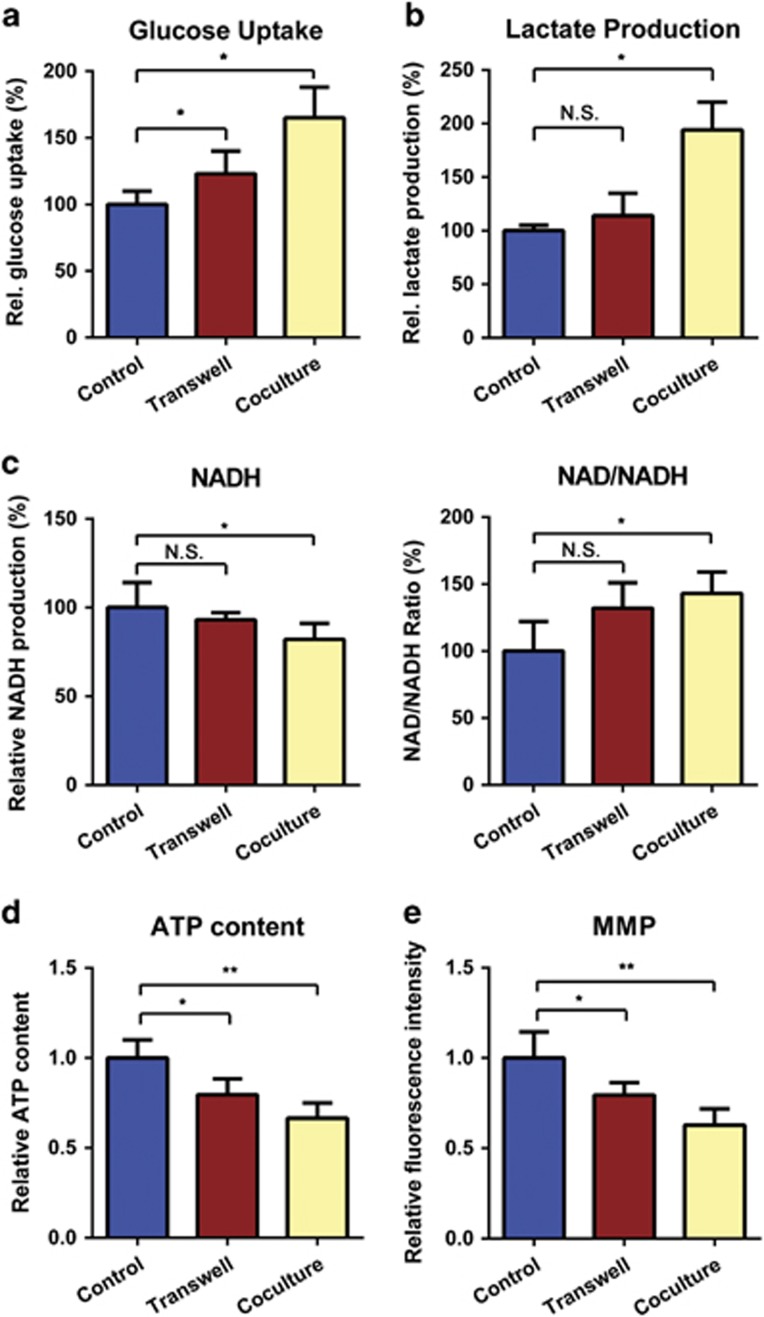
MSCs switch the mitochondrial metabolism of T-ALL cells toward the glycolytic phenotype. (**a** and **b**) The glucose uptake and lactate production capacity were compared in T-ALL cells cultured alone or with MSCs in the Transwell or direct coculture systems. (**c**) Intracellular NADH production and the NAD+/NADH ratio were determined in T-ALL cells subjected to the three culture models. (**d** and **e**) Cellular ATP levels and MMP were most strongly decreased in T-ALL cells directly cocultured with MSCs, compared with cells mono-cultured in suspension. All data above are represented as the mean±S.E.M. of three independent experiments (*P<0.05; **P<0.01; *t*-test)

Under physiological conditions, the mitochondrial respiratory chain is the main site of ROS generation and adenosine triphosphate (ATP) production.^22^ As the metabolic center, mitochondrial metabolic alterations and oxidative stress were widely investigated.^14, 23, 24^ We thus examined whether MSCs regulate energy metabolism in T-ALL cells. Our experiments revealed that glucose uptake and lactate production were significantly elevated in T-ALL cells directly cocultured with MSCs (Figures 3a and b). In addition, the nicotinamide adenine dinucleotide (NADH) level, ATP production and mitochondrial membrane potential (MMP) were decreased in directly cocultured T-ALL cells, whereas the NAD+/NADH ratio was increased (Figures 3c and e). Besides, the mitochondrial mass detected no significant changes after coculture, indicating that the apparent metabolic alterations did not result from the variation in mitochondrial abundance (Supplementary Figure 5). However, the metabolic changes detected in indirectly cocultured T-ALL cells were not that obvious as those directly cocultured (Figures 3a and c). Together, our results show that T-ALL cells display a pro-glycolytic phenotype after direct interaction with MSCs.

### Exposure to MSCs alters the mitochondrial dynamics of T-ALL cells

**Figure 4:**
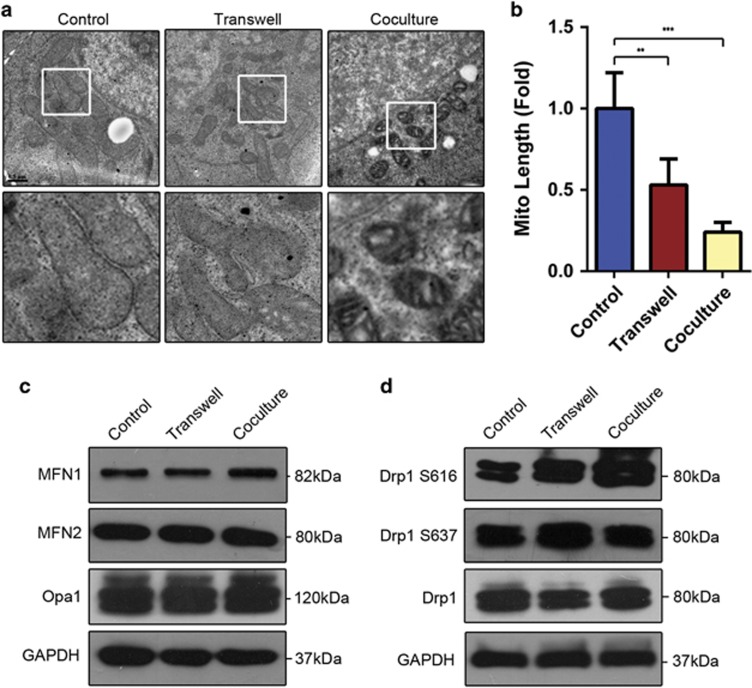
The mitochondrial dynamics of T-ALL cells are altered following direct coculture with MSCs. (**a**) Transmission electron microscopy was used to image mitochondria in primary T-ALL cells cultured with or without MSCs. Scale bars, 0.5 *μ*m. (**b**) Mitochondrial lengths (at least 50 per experiment) were calculated. (**c**) The expression levels of factors known to be associated with mitochondrial dynamics were detected by western blot analysis. (**d**) Immunoblot analysis of the activating phosphorylation (p-Ser616) and repressive phosphorylation (p-Ser637) of Drp1 in primary T-ALL cells cultured with or without MSCs. Data are presented as the mean±S.E.M. (**P<0.01; ***P<0.001; *t*-test)

Mitochondria undergo changes in shape because of constant fusion and fission.^25, 26^ Here, we used electron microscopy to investigate the mitochondrial dynamics in primary T-ALL cells with and without MSCs coculture. We observed the short and fragmented mitochondria in MSC-cocultured T-ALL cells, whereas those in mono-cultured in suspension exhibited elongated and tubular shape (Figures 4a and b).

Among the numerous proteins and pathways known to regulate the dynamic behavior of mitochondria,^27^ Drp1 is the major mediator of mitochondrial fission, whereas mitofusin 1 (MFN1), mitofusin 2 (MFN2) and optic atrophy 1 (OPA1) participate in mitochondrial fusion.^28^ In an effort to account for the observed differences in mitochondrial length, we explored the expression of these key mediators in primary T-ALL cells. However, the qRT-PCR and immunoblotting experiments failed to reveal any significant changes in the expression levels of mitochondrial fusion proteins in T-ALL cells cultured with and without MSCs (Figure 4c and Supplementary Figure 6A). We also detected no consistent differences of mitochondrial fission mediator, Drp1, expression between control and coculture groups, indicating possible regulation at the post-translational levels. (Figure 4d and Supplementary Figure 6A). Post-translational regulations are known to influence mitochondrial dynamics, among which phosphorylation of Drp1 S616 promotes mitochondrial fission, whereas phosphorylation of Drp1 S637 induces mitochondrial fusion.^29^ Thus, we determined the levels of Drp1 phosphorylated at S616 and S637 in primary T-ALL cells cultured with or without MSCs. Interestingly, the level of Drp1 S616 phosphorylation in primary T-ALL cells was strikingly enhanced by MSCs coculture, whereas Drp1 S637 phosphorylation was not significantly altered (Figure 4d). Together, these results show that MSCs influence the mitochondrial dynamics through the regulation of Drp1 phosphorylation in primary T-ALL cells.

### Mutation of Drp1 alters mitochondrial dynamics, ROS generation and the chemoresistance capacity of T-ALL cells

**Figure 5:**
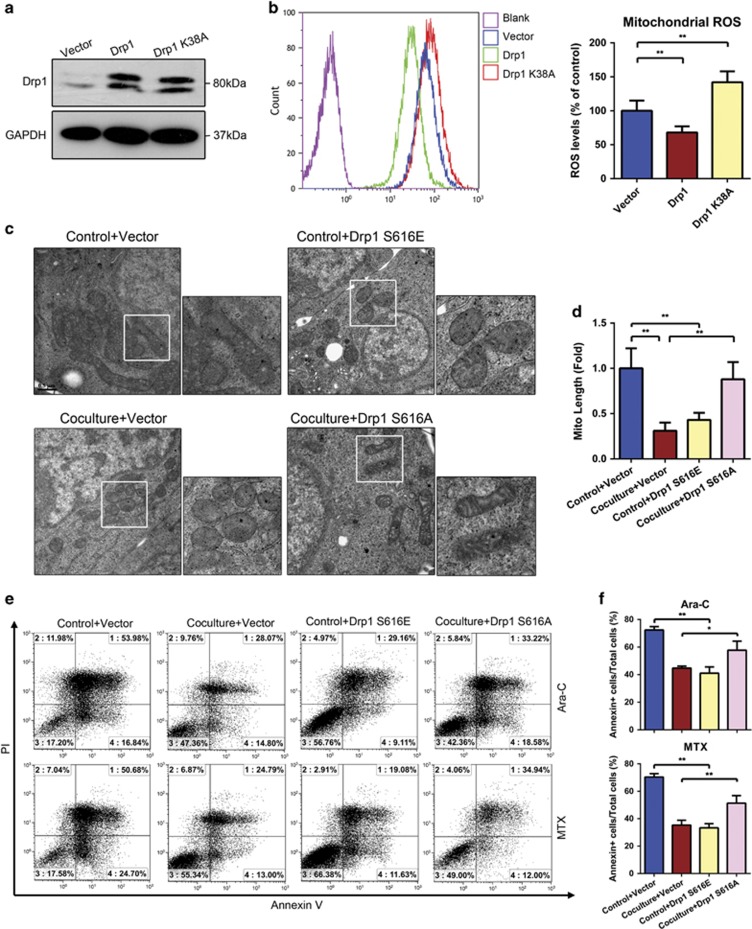
Expression of mutated Drp1 alters mitochondrial dynamics and ROS generation in T-ALL cells, thereby influencing their chemoresistance. (**a**) Immunoblotting analysis of lysates from Jurkat cells overexpressing wild-type Drp1 or the Drp1 K38A mutant. (**b**) NTC, Drp1-overexpressing and Drp1 K38A mutant-expressing Jurkat cells were assessed for mitochondrial ROS generation. (**c**) Transmission electron microscopy was used to visualize mitochondria in primary T-ALL cells expressing Drp1 S616A or Drp1 S616E. Scale bars, 0.5 *μ*m. (**d**) Mitochondrial lengths were calculated for each sample. (**e**) Primary T-ALL cells overexpressing Drp1 S616A or Drp1 S616E were exposed to Ara-C/MTX, and apoptosis was determined using Annexin V/PI staining and FACS. (**f**) The percentages of Annexin V-positive cells were calculated. Data above are presented as the mean±S.E.M. of three independent experiments (*P<0.05; **P<0.01; *t*-test)

To further examine whether Drp1-mediated mitochondrial fission is necessary for the mitochondrial fragmentation and mitochondrial ROS generation observed in T-ALL cells cocultured with MSCs, we assessed the morphologies and ROS levels of mono-cultured Jurkat cells overexpressing wild-type and K38A mutant Drp1 (which inhibits mitochondrial fission).^30^ Immunoblotting confirmed the expression of Drp1 or Drp1 K38A vector in transfected cells (Figure 5a). Meanwhile, transfection of these two vectors in Jurkat cells did not influence the survival of T-ALL cells no matter cultured alone or cocultured with MSCs (Supplementary Figure 7). As expected, the overexpression of Drp1 or Drp1 K38A in Jurkat cells potently induced remodeling of mitochondria (Supplementary Figure 8). The mitochondrial ROS levels were decreased in Drp1-overexpressing cells, whereas those in Drp1 K38A-expressing cells remained high (Figures 5b and c).

As we found that Drp1 S616 in T-ALL cells was phosphorylated after cocultured with MSCs, we continued to assess the potential involvement of Drp1 phosphorylation. Primary T-ALL cells were transfected with vector encoding Drp1 S616A (a non-phosphorylatable mutant) or Drp1 S616E (a phosphomimetic mutant; Supplementary Figure 9)^31^ and subjected to transmission electron microscopy and MitoSOX staining. Our results revealed that overexpression of Drp1 S616E increased mitochondrial fragmentation and decreased mitochondrial ROS levels, whereas overexpression of Drp1 S616A increased the numbers of elongated mitochondria and the levels of mitochondrial ROS in cells directly cocultured with MSCs (Figures 5c, d, Supplementary Figures 10A and 10B). We next determined whether the alterations of mitochondrial morphology induced by the Drp1 mutant could phenocopy the altered mitochondrial metabolism we observed in MSC-cocultured T-ALL cells. Indeed, expression of the phosphomimetic Drp1 mutant increased glucose uptake and lactate production, decreased ATP production and MMP levels; whereas transfection of the Drp1 non-phosphorylatable mutant exhibited the reverse effects (Supplementary Figures 10C-10F). Finally, we investigated the chemoresistance of T-ALL cells expressing Drp1 S616E or Drp1 S616A, and found that Drp1 S616E prevented apoptosis, whereas Drp1 S616A promoted the cytotoxicity of Ara-c/MTX (Figures 5e and f). Taken together, our results show that Drp1 mutants can alter mitochondrial dynamics and ROS generation, thereby influencing T-ALL cells chemoresistance.

### MAPK/ERK activates Drp1 S616 and enhances the chemoresistance of T- ALL cells

**Figure 6:**
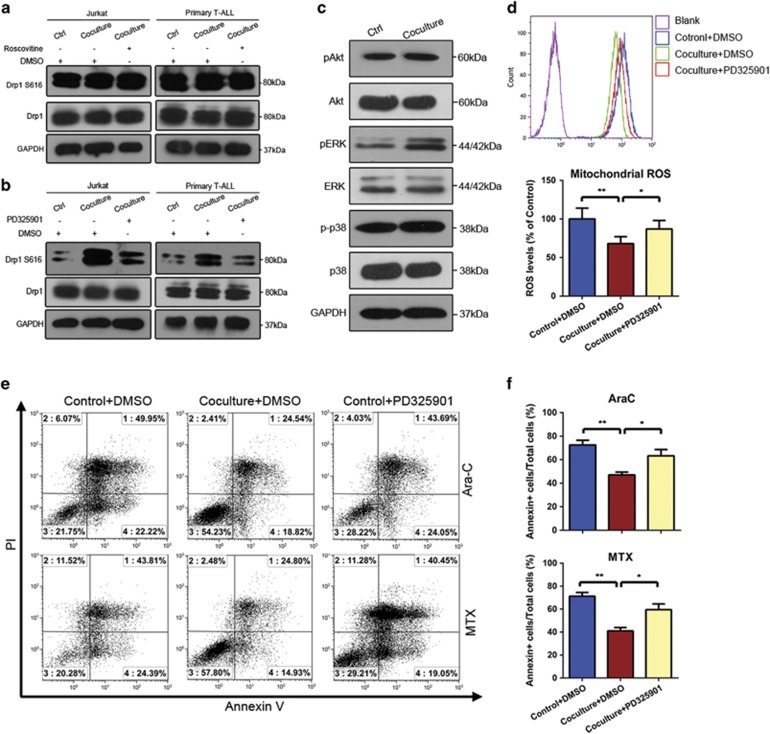
MAPK/ERK signaling triggers the activating phosphorylation of Drp1 at S616, thereby enhancing the chemoresistance of T- ALL cells. (**a** and **b**) Jurkat and primary T-ALL cells were treated with the pan Cdk inhibitor, roscovitine, and MAPK/ERK inhibitor, PD325901. Cell lysates were analyzed by immunoblotting with the indicated antibodies. (**c**) Western blotting analysis of MAPK pathway components in Jurkat and primary T-ALL cells subjected to direct coculture with MSCs. (**d**) Primary T-ALL cells were pretreated with PD325901 and then subjected to direct cocultured with or without MSCs. Statistical analyses of mitochondrial ROS generation. (**e**) Apoptosis of primary T-ALL was measured by Annexin V/PI staining and FACS. (**f**) The percentage of apoptotic cells in each group was analyzed and graphed. Data above are presented as the mean±S.E.M. of three independent experiments (*P<0.05; **P<0.01; *t*-test)

To determine the mechanisms underlying Drp1 activation, we tested candidate kinases in Jurkat and primary T-ALL cells cultured with or without MSCs. Cyclin-dependent kinase (Cdk) family and mitogen-activated protein kinase (MAPK)/ERK were previously reported to phosphorylate Drp1 at Ser616.^32^ We found that the pan Cdk inhibitor, roscovitine, did not alter Drp1 S616 phosphorylation. In contrast, the ERK inhibitor, PD325901 (10 *μ*M), significantly repressed Drp1 S616 phosphorylation in our systems (Figures 6a and b). Moreover, we explored the MAPK pathway activity in primary T-ALL cells directly cocultured with or without MSCs. We found that ERK phosphorylation was elevated, whereas there was no significant changes in the phosphorylation levels of phosphatidylinositol 3-kinase/Akt or p38 MAPK (Figure 6c). These results suggest that Drp1 S616 phosphorylation in T-ALL cells, and thus the protective effects of MSCs on these cells, could be mediated by ERK pathway activation.

Accordingly, we examined the involvement of ERK phosphorylation in the MSC-mediated cell survival of T-ALL cells exposed to Ara-C/MTX. First, inhibition of ERK activity had no effects on viability of T-ALL cells when cultured alone or cocultured with MSCs (Supplementary Figure 11). We found that the PD325901-mediated inhibition of ERK activation increased mitochondrial ROS levels (Figure 6d) and abolished the capacity of MSCs to protect T-ALL cells against Ara-C/MTX-induced apoptosis (Figures 6e and f). Collectively, these results indicate that the MAPK/ERK pathway activates Drp1 in T-ALL cells, contributing to the MSC-mediated survival.

## Discussion

Many studies have focused on the roles of components in the bone marrow microenvironment, especially MSCs, in promoting leukemia chemoresistance. However, the underlying mechanisms are not well understood, especially in terms of the effects on mitochondria. Here, we report that: (i) the pro-survival effects of direct coculture with MSCs are more superior to those in Transwell models; (ii) MAPK/ERK signaling pathway is remarkably activated in T-ALL cells cocultured with MSCs; and (iii) this signaling pathway promotes the phosphorylation of Drp1 at S616 and alters mitochondrial metabolism.

SFM-DR and CAM-DR are two main strategies that responsible for MSC-mediated leukemia chemoresistance. The models of direct contact between leukemia cells and MSCs presented both SFM-DR and CAM-DR, provided opportunities of substance (such as organelles) and signaling pathways communication, which were more likely to mimic the bone marrow microenvironment *in vivo*. However, the Transwell models were just used to study the complex reciprocal regulation of soluble factor expression in MSCs and leukemia cells cross-talk.^33, 34^ Combined with the two drug resistance manners, the direct coculture systems thus exhibited more solid pro-survival effects than Transwell models did, which was consistent with our results.

Many studies have focused on the protective roles of mitochondrial metabolism on tumor cells survival against cytotoxicity of chemotherapeutic agents. Unlike normal differentiated cells, which rely on mitochondrial oxidative phosphorylation, most cancer cells depend on aerobic glycolysis to provide the metabolic substances to meet their proliferative requirements; this is termed 'the Warburg effect'.^35, 36^ Saito *et al.*^37^ found adenosine 5'-monophosphate (AMP)-activated protein kinase signaling protects leukemia-initiating cells by increasing glucose uptake, shifting the metabolism toward glycolysis and reducing intracellular ROS levels in acute myeloid leukemia (AML). Moreover, the recent review suggested that metabolic switch of solid tumors toward glycolysis resulted from environmental selection, which was positively related to cell survival.^38^ Samudio *et al.*^39, 40^ found that the coculture of leukemia cells with MSCs increased the Warburg effect (pro-glycolysis) by upregulating the expression of uncoupling protein 2, which was known to uncouple oxidative phosphorylation and thus reduced mitochondrial ROS generation in leukemia cells. The metabolic phenotype alterations observed in our study were mostly consistent with these literatures. Meanwhile, the glycolytic alterations in Transwell assays were not as remarkable as those in coculture assays, indicating that the metabolic switch may account for chemoresistance.

In addition, we observed that the mitochondrial dynamics of T-ALL cells were altered by MSCs coculture. T-ALL cells cultured in both Transwell and direct contact systems were presented with shorter mitochondria when compared with mono-cultured cells. This was caused by a remarkable elevation of the activating phosphorylation of Drp1 at S616, which is a key mediating event in mitochondrial fission.^27, 41^ T-ALL cells overexpressing wild-type Drp1 or Drp1 S616E exhibited fragmented mitochondria, decreased mitochondrial ROS levels, a pro-glycolytic metabolism shift and increased drug resistance. Several emerging studies focused on the relationship between mitochondrial dynamics and cell fate determination.^42, 43, 44, 45, 46, 47^ For example, Serasinghe *et al.*^48^ reported that mitochondrial division and pro-glycolytic metabolism switch mediated by Drp1 S616 phosphorylation were necessary in RAS-induced transformation. Moreover, disruption of Drp1 activity was shown to trigger mitochondrial fusion and inhibit tumor growth.^49^ Collectively, these results and our present findings indicate that Drp1-mediated mitochondrial dynamics are linked with the switch of mitochondrial glycolysis.

Recently, Moschoi *et al.*^50^ have demonstrated that MSCs protected AML from chemotherapy via transferring functional mitochondria to AML cells characterized by increase in mitochondrial ATP generation *in vivo* and *in vitro*. However, we found that the mitochondrial transferring process was not that obvious from MSCs to T-ALL cells in our study (data not shown). It may suggest that not all cells were recipients for mitochondrial transfer. In conclusion, we herein show for the first time that the ability of MSCs to protect T-ALL cells against chemotherapeutic cytotoxicity is partially dependent on decreased mitochondrial ROS levels and a pro-glycolytic metabolic switch. Moreover, these protective effects are accompanied with a mitochondrial fragmentation process, which was governed by the ERK-mediated activating phosphorylation of Drp1. Thus, disruption of leukemia cells/stromal interactions and targeting mitochondrial dynamics may provide a novel strategy that could be combined with conventional chemotherapeutic agents for the T-ALL treatment.

## Materials and Methods

### Cell culture and primary T-ALL blasts

The human T-ALL cell line, Jurkat, was obtained from the Cell Bank of the Chinese Academic of Science (Shanghai, China). Cells were maintained in RPMI 1640 (Hyclone, Logan, UT, USA) supplemented with 10% fetal bovine serum (FBS; Gibco, Grand Island, NY, USA), and 100 units/ml penicillin and streptomycin (Sigma, St. Louis, MO, USA).

The 10 enrolled T-ALL patients were previously untreated and newly diagnosed at the Department of Pediatrics, Sun Yat-Sen Memorial Hospital, Sun Yat-Sen University (Guangzhou, China).All human bone marrow samples were obtained with written informed consent. Primary CD3^+^ T-ALL cells were isolated from the bone marrow samples through density gradient centrifugation on standard Ficoll-HyPaque, and subjected to fluorescence-activated cell sorting (FACS; BD Bioscience Influx, Franklin Lakes, NJ, USA).

For collection of MSCs, bone marrow aspirates were obtained with informed consent from healthy volunteers, and MSCs were isolated and the characteristics of MSCs were analyzed as we previously described.^51, 52^

For the culture models, (1) T-ALL cells (5 × 10^5^/ml) were mono-cultured in suspension; (2) T-ALL cells (5 × 10^5^/ml) were cocultured directly with a feeder layer of MSCs (5 × 10^4^/ml); (3) T-ALL cells (5 × 10^5^ cells/ml) were seeded in the top chambers, which were inserted into the bottom wells of the 24-well plates with pre-seeded MSCs (5 × 10^4^ cells/ml). After mono-culture or coculture for 2 days, T-ALL cells were then treated with various compounds (Ara-C or MTX) and the relative measurements were performed after additional coculture for 2 days.

### Reagents and antibodies

Ara-C and MTX were purchased from Pharmacia Pty Ltd (NSW, Australia) and Calbiochem (San Diego, CA, USA), respectively. MitoTEMPO was obtained from Santa Cruz Biotechnology (Dallas, TX, USA). The ERK inhibitor, PD325901, was purchased from Millipore Calbiochem (San Diego, CA, USA), resuspended in dimethyl sulfoxide (Sigma) at a stock concentration of 10 mM, and diluted to a final concentration of 10 *μ*M. The pan Cdk inhibitor, roscovitine, was purchased from Selleckchem (Radnor, PA, USA) and used at a final concentration of 0.4 *μ*M. The utilized primary and secondary antibodies are listed in the Supplementary Table 1.

### Annexin V/PI flow cytometric analysis

Cells were treated with Ara-C or MTX for 48 h, harvested, and resuspended in 500 *μ*l of binding buffer in Annexin V/propidium iodide (PI) assay kit (BIOSCI BIOTECH, Shanghai, China). The samples were then incubated for 15 min at room temperature in the dark with PI and FITC-conjugated Annexin V, and the apoptotic population was immediately evaluated by flow cytometry (FACScan; Becton Dickinson, San Diego, CA, USA). Annexin V+/PI− cells were considered early apoptotic cells, whereas Annexin V+/PI+ cells were considered late apoptotic cells.

### ROS assessment

The levels of total intracellular ROS and mitochondrial ROS were detected using the fluorescent probes, CellROX Deep Red and MitoSOX (both from Molecular Probes, Life Technologies, Carlsbad, CA, USA), respectively, and fluorescent intensity was measured by flow cytometry. The levels of extracellular ROS were determined with the chemiluminescent probe, Diogenes (National Diagnostics, Atlanta, GA, USA), which was applied per the manufacturer's instructions.

### RNA isolation and qRT-PCR

Cells were harvested, total mRNA was isolated using the TRIzol Reagent (Invitrogen, Carlsbad, CA, USA), and reverse transcription was performed using a QuantiTect Reverse Transcription kit (Qiagen, Valencia, CA, USA) according to the manufacturer's instructions. qRT-PCR was performed with SYBR Green qPCR SuperMix (Roche, Indianapolis, IN, USA) and a Light Cycler 480 Detection System (Roche). Target mRNA levels were normalized with respect to those of *β*-actin. The primer sequences used for qRT-PCR are listed in the Supplementary Table 2.

### Cell viability assays

Cell viability was determined using a CCK-8 assay kit (Dojin Laboratories, Kumamoto, Japan) according to the manufacturer's instructions. Briefly, cells were plated to a 96-well plate (5 × 10^5^ cells/ml; 100 *μ*l/well) and incubated at 37 °C under 5% CO_2_.The CCK-8 solution (10 *μ*l) was added, the samples were incubated for 4 h at 37 °C, and absorbance at 450 nm was quantified using an automated enzyme-linked immunosorbent assay reader (Tecan, Salzburg, Austria).

### Cytotoxicity assay

Cells were exposed to chemotherapeutic drugs for 48 h and stained using a LIVE/DEAD^®^ viability/cytotoxicity assay kit (Invitrogen). The cells were further stained with Hoechst33342 and mounted on a slide. The percentages of dead cells (EthD-1 positive) were determined for high-power fields and calculated using the ImageJ software (National Institute of Health, Bethesda, MD, USA). A minimum of 500 cells were counted per experiment.

### ATP content detection

The cellular ATP content was determined using a CellTiter-Glo Luminescent Cell Viability Assay kit (Promega, Madison, WI, USA), as described by the manufacturer. Briefly, cells (1 × 10^5^) were plated in a 96-well plate, mixed with an equal volume of reagent for 2 min, and incubated at room temperature for 10 min. Luminescence was recorded using a GENios Plus fluorescence microplate reader (TECAM Company, Switzerland).

### Detection of MMP

The MMP was measured using the fluorescent probe, MitoTracker (Invitrogen), according to the manufacturer's recommendations. Briefly, samples were stained with MitoTracker at 37 °C for 30 min and washed twice, and the fluorescent intensity was quantified by flow cytometry.

### Western blotting

Cells subjected to the indicated treatments were collected, lysed in 1 × RIPA buffer, and centrifuged at 15 000 *g* for 5 min at 4 °C. The supernatant was collected as the total cell lysate. Equal amounts of protein were resolved by SDS-PAGE and electrotransferred to a 0.45-*μ*m-porepolyvinylidene difluoride membrane (Millipore, Bedford, MA, USA). The membrane was blocked with 5% milk for 1 h, incubated overnight with the relevant primary antibodies, and then incubated with horseradish peroxidase-conjugated secondary antibodies at room temperature for 1 h. The immunoreactive bands were detected with an enhanced chemiluminescence kit (Millipore).

### Metabolism detection assays

The NAD+/NADH ratio, lactate production and intracellular glucose uptake were measured using the relevant detection kits (all from BioVision, Milpitas, CA, USA) according to the manufacturer's directions.

### Transmission electron microscopy

The samples were fixed in 2.5% glutaraldehyde (pH7.4) for 2 h, post-fixed with 1% osmium tetroxide for 1 h, washed, dehydrated through an ethanol series (30, 50, 70 and 95%, 5 min per step), embedded and polymerized at 60 °C for 48 h. Ultrathin sections (85 nm) were cut using a diamond knife, stained with uranyl acetate and lead citrate, and observed using a Tecnai G2 Spirit Twin transmission electron microscope (FEI Company, Eindhoven, The Netherlands) operated at 80 kV.

### Transfection of vectors

Drp1-overexpressing (plasmid #45160) and Drp1 K38A-expressing (plasmid #45161) vectors were purchased from Addgene (Cambridge, MA, USA). TheS616E and S616A mutants of Drp1 were generated using overlap PCR assays described as Supplementary Figure 7 in details. The utilized primer sequences were as follows: Drp1 S616E forward, 5′-ATTCCAATTATGCCAGCCGAGCCACAAAAAGGTCATGCCGT-3′ and reverse, 5′-ACGGCATGACCTTTTTGTGGCTCGGCTGGCATAATTGGAAT-3′; and Drp1 S616A forward, 5′-GTTCCTGTTGCACGAAAACTAGCTGCTCGGGAAC-3′ and reverse, 5′-GTTCCCGAGCAGCTAGTTTTCGTGCAACAGGAAC-3′. Cells were transfected with these plasmids using the X-treme GENE HP reagent (Roche) according to the manufacturer's instructions.

### Statistical analyses

All data are expressed as the mean±S.E.M. from at least three independent experiments. Comparisons among groups were performed using one-way analysis of variance (ANOVA) or Student's *t*-test. CCK-8 assays were tested using repeated ANOVA followed by a post-hoc test. The statistical package R (version 3.1.1, University of Auckland, Auckland, New Zealand) was used for all analyses. A two-sided *P*-value <0.05 was considered to be statistically significant.

## Abbreviations

Akt: serine/threonine kinase
AML: acute myeloid leukemia
ANOVA: analysis of variance
Ara-C: cytosine arabinoside
ATP: adenosine triphosphate
CAM-DR: cell adhesion-mediated drug resistance
CCK-8: cell counting kit-8
Cdk: cyclin-dependent kinases
CLL: chronic lymphocytic leukemia
Drp1: dynamin-related protein 1
EMDR: environment-mediated drug resistance
ERK: extracellular signal-regulated kinase
FACS: fluorescence-activated cell sorting
FBS: fetal bovine serum
MAPK: mitogen-activated protein kinase
MFN1: mitofusin 1
MFN2: mitofusin 2
MMP: mitochondrial membrane potential
MRD: minimal residual disease
MSC: mesenchymal stem cell
MTX: methotrexate
NADH: nicotinamide adenine dinucleotide
Nox: NADPH oxidase
OPA1: optic atrophy 1
PI: propidium iodide
qRT-PCR: quantitative real-time polymerase chain reaction
ROS: reactive oxygen species
SFM-DR: soluble factor-mediated drug resistance
T-ALL: T-cell acute lymphoblastic leukemia
