# Supplementary material for: ERK/Drp1-dependent mitochondrial fission is involved in the MSC-induced drug resistance of T-cell acute lymphoblastic leukemia cells
Source: Cell Death Dis. 2016 Nov 10;7(11):e2459–. doi: 10.1038/cddis.2016.370 (PMC5260898; doi:10.1038/cddis.2016.370)
Supplement: Supplementary Information [file cddis2016370x1.docx]

**SUPPLEMENTARY INFORMATION**

**ERK/Drp1-dependent mitochondrial fission is involved in the MSC-induced drug resistance of T-cell acute lymphoblastic leukemia cells**

Jianye Cai, Jiancheng Wang, Yinong Huang, Haoxiang Wu, Ting Xia, Jiaqi Xiao, Xiaoyong Chen, Hongyu Li, Yuan Qiu, Yingnan Wang, Tao Wang, Huimin Xia, Qi Zhang & Andy Peng Xiang

**- SUPPLEMENTARY FIGURES 1-11**

**- SUPPLEMENTARY TABLES 1-2**

**Supplementary figures and supplementary figure legends**


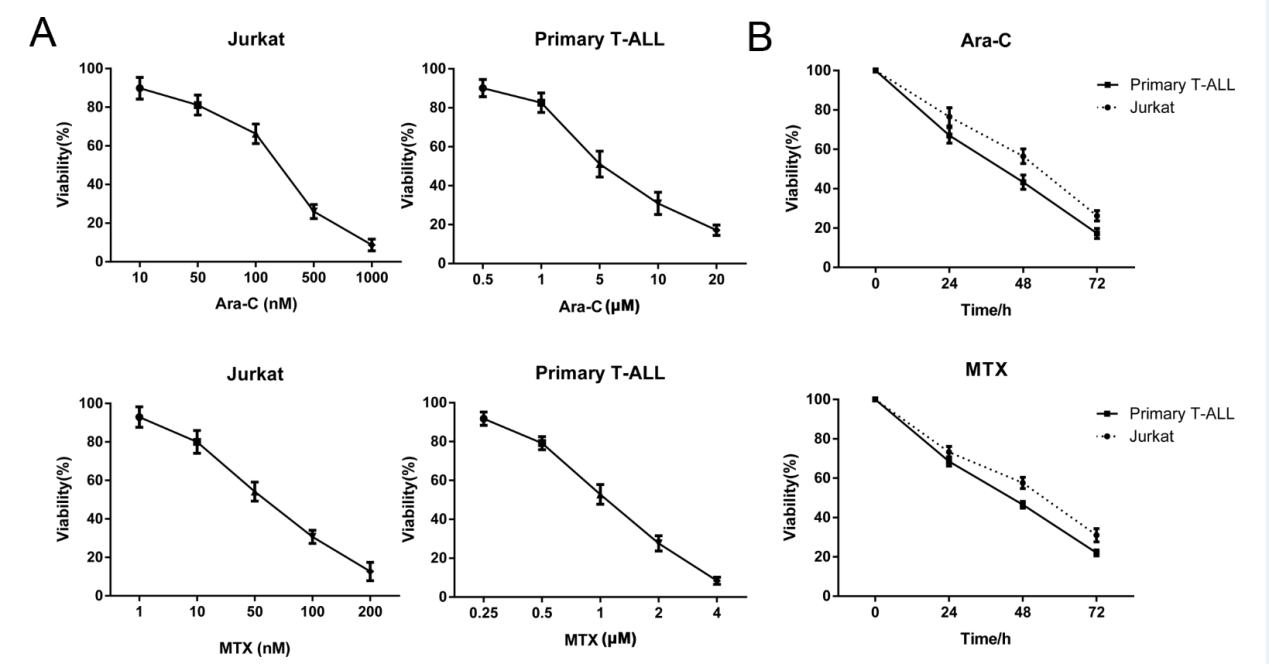


**Supplementary Figure 1. Effect of Ara-C/MTX on cell viabilities in T-ALL cell line and primary T-ALL cells**

(A) Jurkat and primary T-ALL cells were treated with various concentrations of Ara-C or MTX for 48 h, and cell viability was measured using the CCK-8 assay. (B) Jurkat and primary T-ALL cells were treated with Ara-C and MTX (300 nM Ara-C and 100 nM MTX for Jurkat and 6 µM and 1.5 µM for primary T-ALL cells) for different durations, and cell viability was determined.


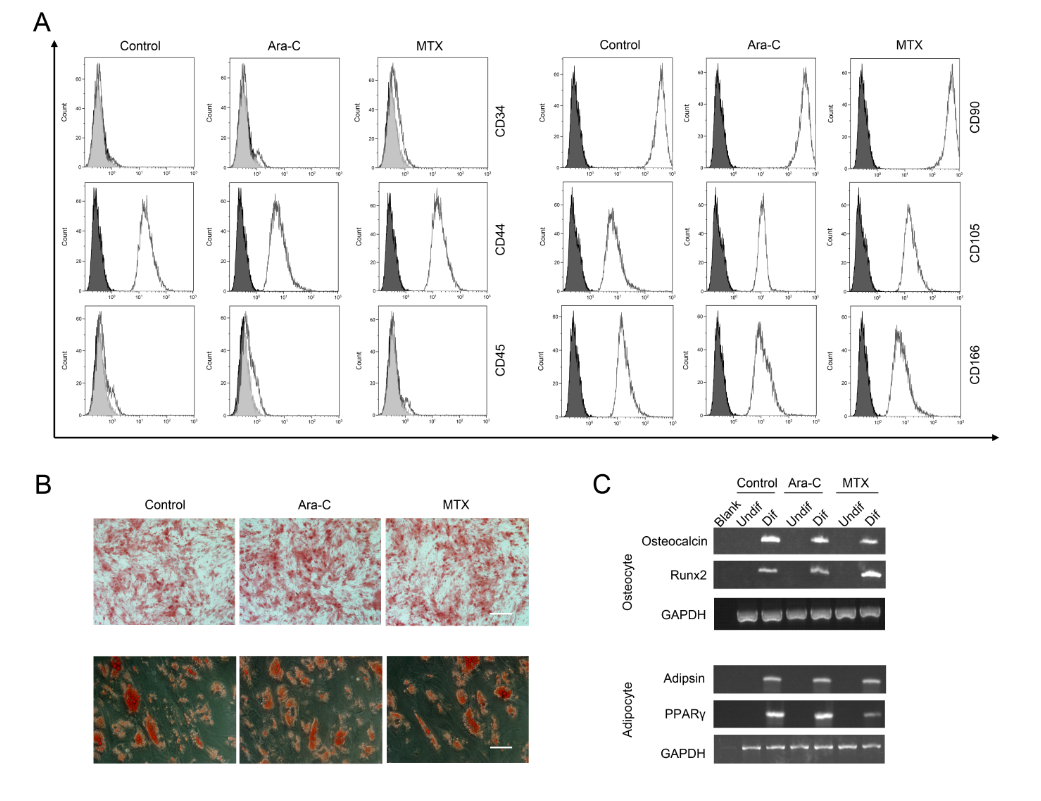


**Supplementary Figure 2: Characterization of cocultured MSCs after treatment of chemotherapeutic agents.**

(A) The expression of surface marker CD34, CD44, CD45, CD90, CD105 and CD166 were detected by flow cytometry in cocultured MSCs treated with or without MTX/Ara-C. (B) Cocultured MSCs were collected and cultured in differentiation medium for 3 weeks. Differentiation of MSCs into osteoblasts and adipocytes were confirmed by Alizarin Red S and Oil Red O staining, respectively. Scale bar, 50 μm. (C) Osteogenic and adipogenic markers of differentiated MSCs were analyzed by RT-PCR.


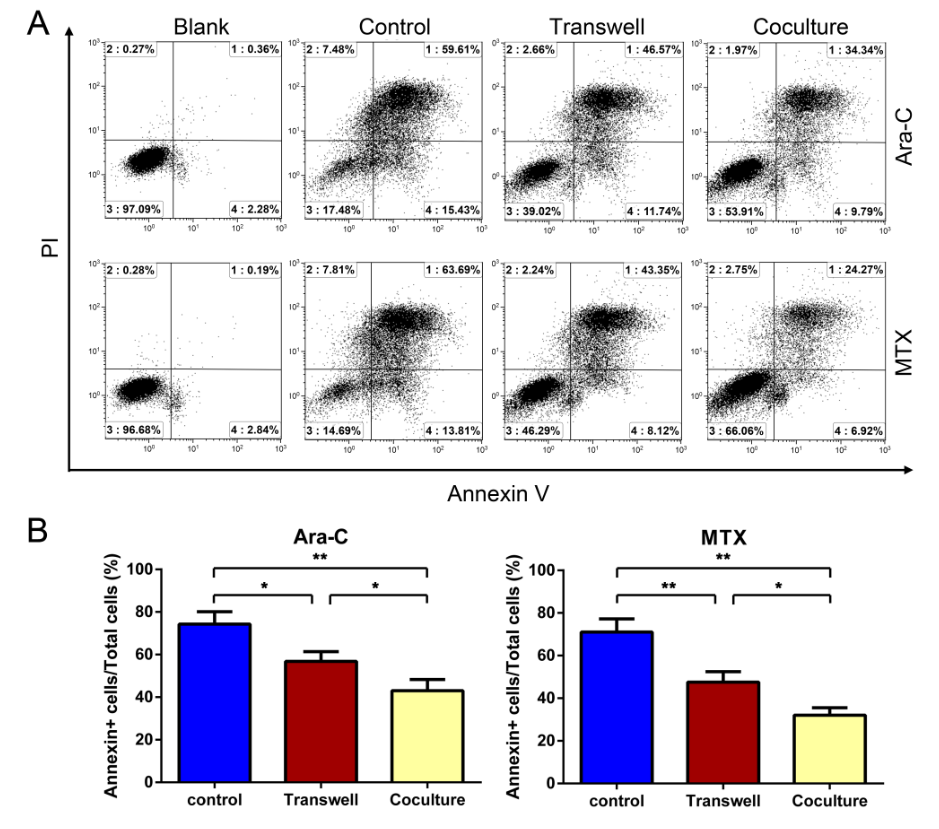


**Supplementary Figure 3. MSCs promote T-ALL cells survival in both Transwell and direct coculture models**

(A) Primary T-ALL cells were isolated, cultured with or without MSCs and treated with Ara-C or MTX for 48 h, and apoptosis was measured by Annexin V/PI staining and flow cytometry. (B) Histograms were quantiﬁed to analyze the percentage of Annexin V-positive cells. Data are presented as the mean ± SEM (n=3) for each group (*p < 0.05; **p < 0.01; t-test).


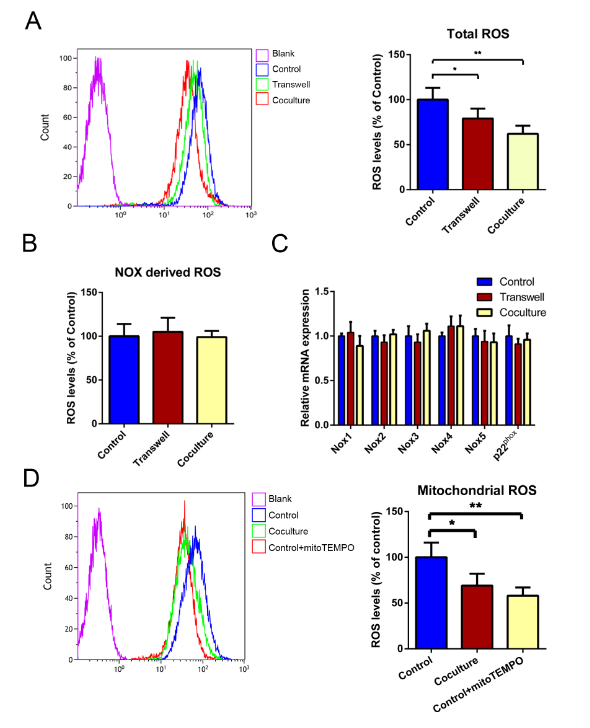


**Supplementary Figure 4. The effect of MSCs on intracellular ROS levels of Jurkat T-ALL cells.**

(A) Density plot analysis of total ROS levels in Jurkat cells mono-cultured in suspension or cocultured with MSCs in Transwell and direct coculture system. Statistical analyses showed that primary T-ALL cells subjected to direct MSC coculture display less total ROS generation than the other two groups. Data are presented as the mean ± SEM (n=3) for each group (*p < 0.05; **p < 0.01; t-test). (B) Total superoxide production was measured in Jurkat cells cultured with or without MSCs. Statistical analyses show that extracellular superoxide generation was not significantly altered by coculture with MSCs. Results are expressed as the mean ± SEM of three independent experiments.(C) Jurkat cells were cultured with or without MSCs, and Nox gene expression was assessed by qRT-PCR. No significant expressional alteration was observed in the tested Nox family members. Data are presented as the mean ± SEM of three independent experiments. (D) Flow cytometry was used to measure the mitochondrial ROS levels of T-ALL cells. Statistical analyses show that treatment with MitoTEMPO (50μM for 6 h) reduces the mitochondrial ROS levels in these cells. Data are presented as the mean ± SEM of three independent experiments (*p < 0.05; **p < 0.01; t-test).


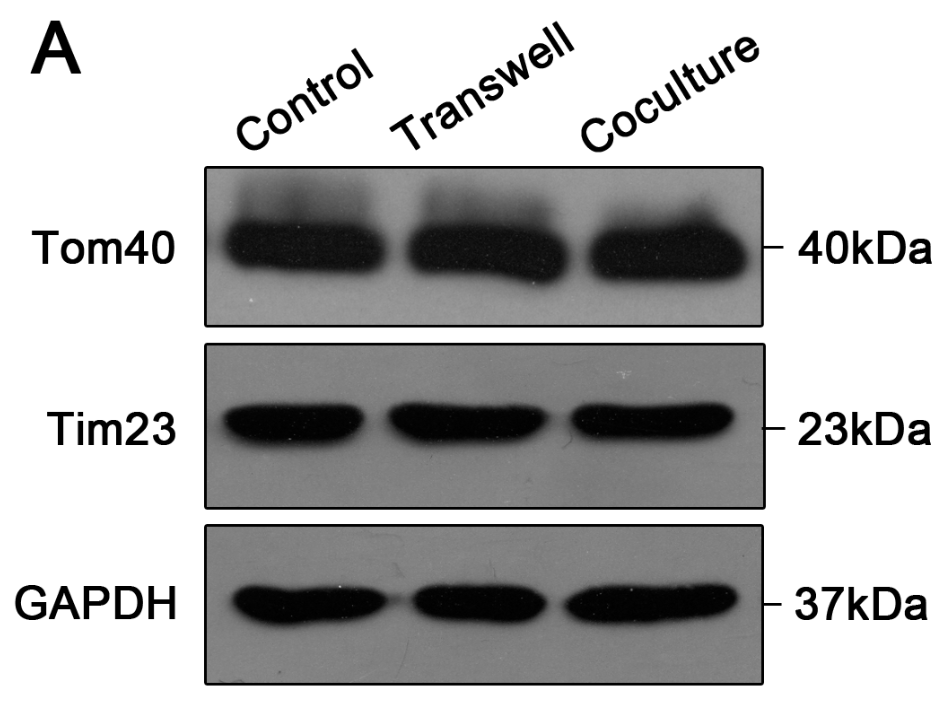


**Supplementary Figure 5.**

(A) Western blots showing that the mitochondrial masses did not significantly differ in T-ALL cells subjected to mono-culture, direct MSC coculture, or indirect MSC coculture.


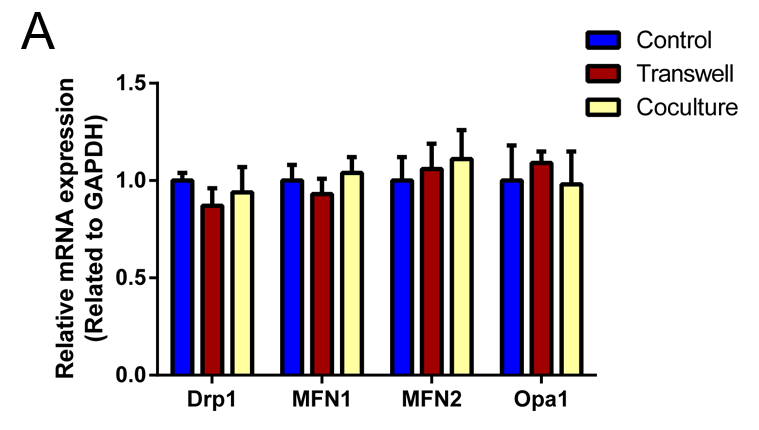


**Supplementary Figure 6.**

(A) The mRNA expression levels of mitochondrial dynamics-related factors were not significantly different in Jurkat cells cultured with or without MSCs


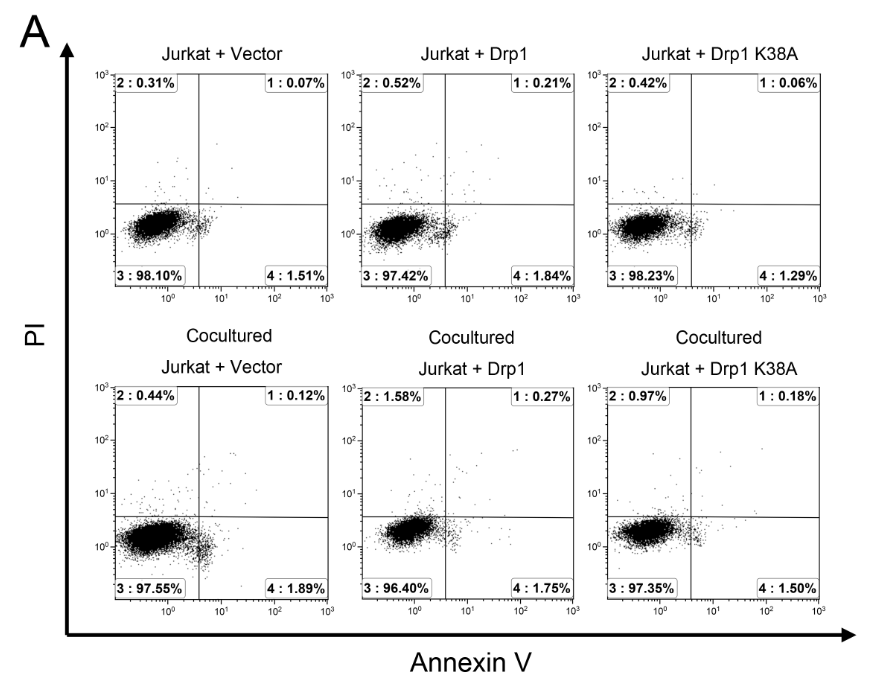


**Supplementary Figure 7.**

(A) Transfection of Drp1 overexpression or Drp1 K38A vectors did not influence the viability of Jurkat cells when cultured alone cocultured with MSCs. Data are means ± SEM of three independent experiments.


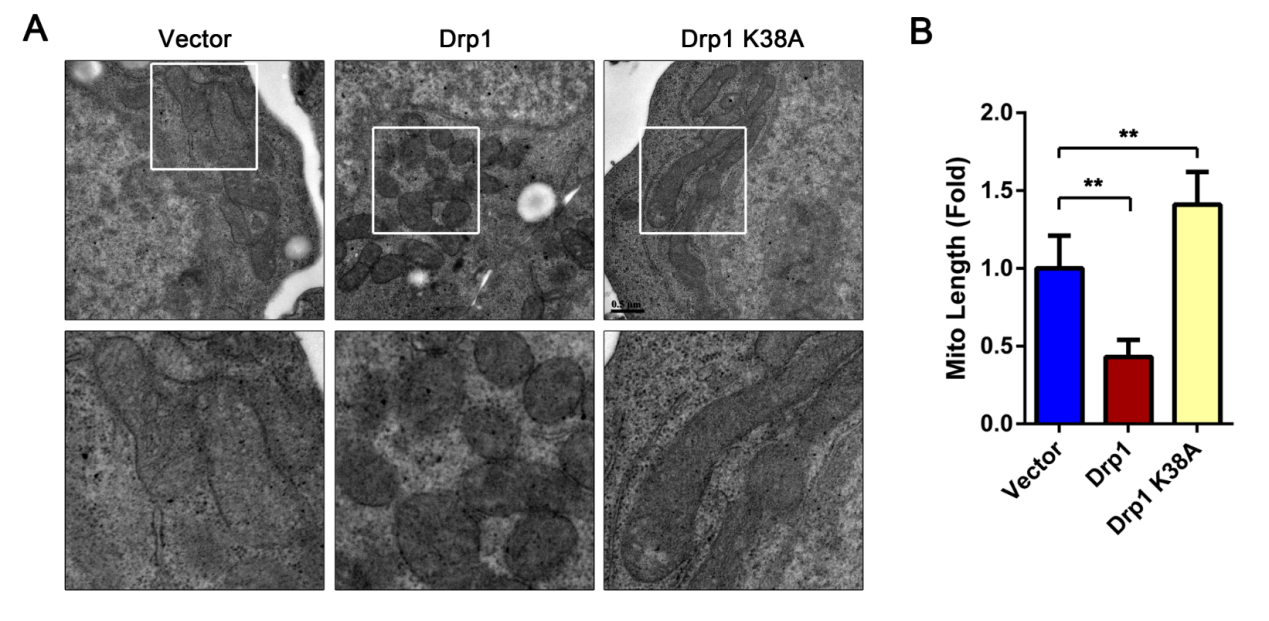


**Supplementary Figure 8. Mitochondrial morphology was influenced after overexpressing Drp1 and Drp1 K38A vectors.**

(A) The mitochondrial dynamics of Jurkat cells with or without overexpression of Drp1 or Drp1 K38A were observed by transmission electron microscopy. Magnified images are shown in insets. Scale bars, 0.5 µm. (B) Mitochondrial length were calculated for at least 50 mitochondria per experiment. Data are presented as mean ± SEM (**p < 0.01; t-test).


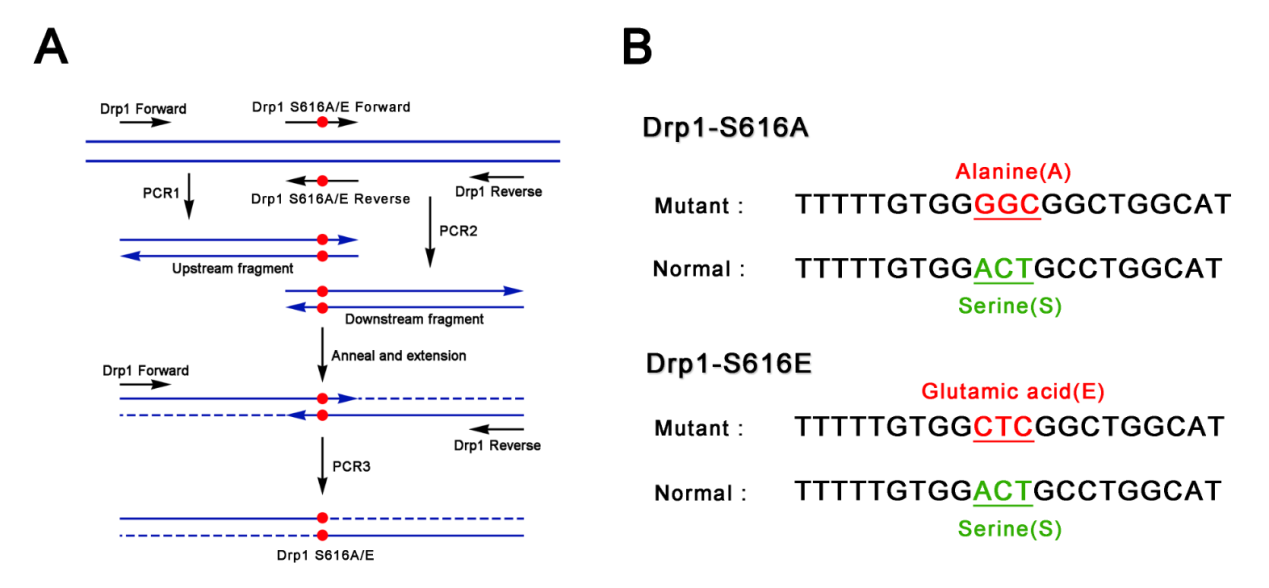


**Supplementary Figure 9. Schematic diagram of PCR and overlap PCR to construct Drp1 mutant vectors, Drp1 S616A and Drp1 S616E**

(A)Two primary PCR products with overlapping ends were synthesized by the first PCR reaction with two pair Drp1 primes containing mutant sites. These two fragments were annealed and extended into full length of Drp1 mutant, followed by subsequent third PCR step to amplify target genes. (B) Sequence of final amplified expression template.


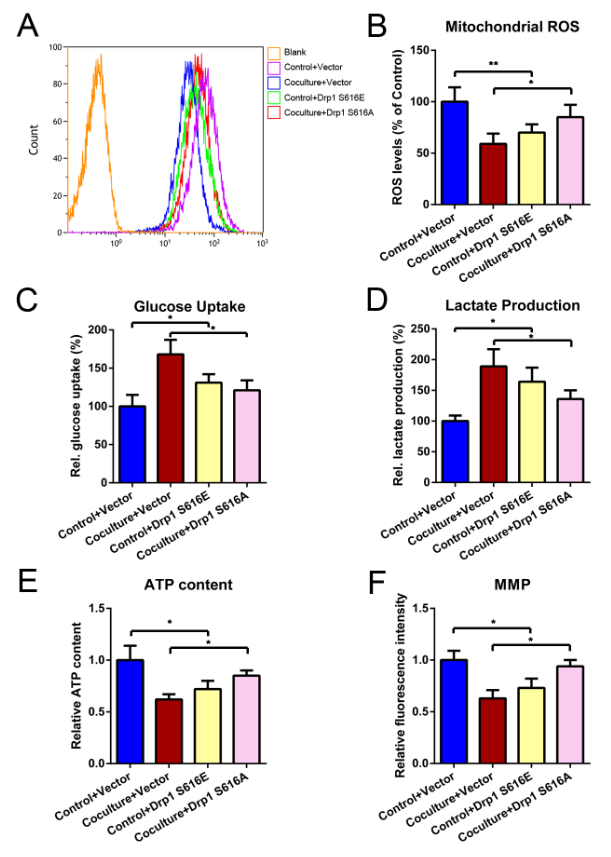


**Supplementary Figure 10. Mitochondrial ROS generation and metabolic phenotype switch caused by alteration of mitochondrial dynamics.**

(A) T-ALL cells were subjected to the indicated treatments, and mitochondrial ROS levels were measured by flow cytometry. (B) Statistical analyses of mitochondrial ROS levels. Data are means ± SEM of three independent experiments (*p < 0.05; **p < 0.01; t-test). (C-F) Glucose uptake, lactate production, ATP content and MMP of T-ALL cells after different treatment were determined as described in Materials and Methods (*p < 0.05; t-test).


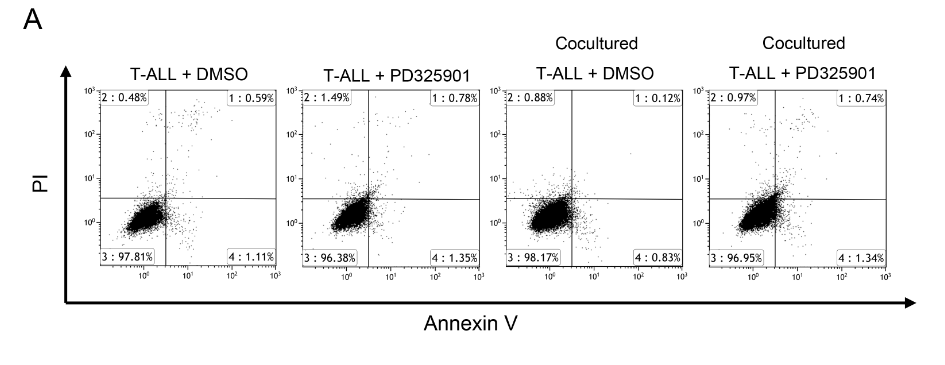


**Supplementary Figure 11.**

(A) Inhibition of ERK activity by PD325901 did not influence the cell viability of T-ALL cells when cultured alone cocultured with MSCs. Data are means ± SEM of three independent experiments.

| **MARKER (SPECIES)** | **DILUTION** | **DISTRIBUTOR/SOURCE (CATALOG NUMBER)** |
| --- | --- | --- |
| **Primary antibody:** |  |  |
| **WB:**  **Tom40 (H300) rabbit IgG**  **Tim23 (C-19) goat IgG** | **1:1000**  **1:1000** | **Santa Cruz (sc-11414)**  **Santa Cruz (sc-13298)** |
| **Drp-1 (H-300) rabbit IgG**  **Phospho-Drp1 (Ser616) (D9A1) rabbit mAb**  **Phospho-Drp1 (Ser637) (D3A4) rabbit mAb** | **1:1000**  **1:1000**  **1:1000** | **Santa Cruz (sc-32898)**  **CST (4494)**  **CST (6319)** |
| **GAPDH (14C10) rabbit Ab**  **MFN1 (D6E2S) rabbit mAb**  **MFN2 (D1E9) rabbit mAb**  **OPA1 antibody (D-9)**  **ERK 1/2 antibody**  **p-ERK (Thr202/Tyr204) antibody**  **Akt (C67E7) rabbit mAb**  **p-Akt rabbit mAb**  **p38MAPK rabbit mAb**  **phopho-p38MAPK (Thr180/Tyr182) Antibody** | **1:2000**  **1:1000**  **1:1000**  **1:1000**  **1:1000**  **1:1000**  **1:1000**  **1:1000**  **1:1000**  **1:1000** | **CST (sc-659)**  **CST (14739)**  **CST (11925)**  **Santa Cruz (sc-393296)**  **CST (9102)**  **CST (9101s)**  **CST (4691s)**  **CST (4046s)**  **CST (9212)**  **CST (9211)** |
| **Secondary antibody:**  **WB:**  **Anti-mouse IgG HRP-linked Ab**  **Anti-rabbit IgG HRP-linked Ab**  **Donkey anti-goat IgG HRP Ab** | **1:5000**  **1:5000**  **1:5000** | **CST (7076)**  **CST (7074)**  **Santa Cruz (sc-2020)** |

**Supplementary Table 1. Antibody used for immunoblotting**

| **Supplementary Table 2. Primer used to amplify the transcripts during real-time quantitative PCR.** | | |
| --- | --- | --- |
| **Gene (human)** | **Sequence (5′ to 3′)** | **Application** |
| **Nox1** | **Upper: ATGATCTGCCTACATACAGC**  **Lower: GGATTTAGCCAAGAACCCC** | **qRT-PCR** |
| **Nox2** | **Upper: ACACATGCCTTTGAGTGGTT**  **Lower: TGTTCCTTTCCTGCATCTGG** | **qRT-PCR** |
| **Nox3** | **Upper: ACCTTCTGTAGAGACCGCTA**  **Lower: CTTGTTGAAATCGCCAGAACC** | **qRT-PCR** |
| **Nox4**  **Nox5** | **Upper: CACCTCTGCCTGTTCATCTG**  **Lower: GGCTCTGCTTAGACACAATCC**  **Upper: CACTGACCCTGCTCATCCA**  **Lower: GCACCCCACTCTGTACCTG** | **qRT-PCR**  **qRT-PCR** |
| **p22^phox^** | **Upper: GCCCATCGAGCCCAAGCC**  **Lower: CTGCTTGATGGTGCCTCCGA** | **qRT-PCR** |
| **Drp1** | **Upper: AAGAACCAACCACAGGCAAC**  **Lower: GTTCACGGCATGACCTTTTT** | **qRT-PCR** |
| **MFN1**  **MFN2**  **OPA1** | **Upper: TTGGAGCGGAGACTTAGCAT**  **Lower: TTCGATCAAGTTCCGGATTC**  **Upper: AGAGGCATCAGTGAGGTGCT**  **Lower: GCAGAACTTTGTCCCAGAGC**  **Upper: GGCCAGCAAGATTAGCTACG**  **Lower: ACAATGTCAGGCACAATCCA** | **qRT-PCR**  **qRT-PCR**  **qRT-PCR** |
| **β-actin** | **Upper: ACTTAGTTGCGTTACACC**  **Lower: AATCCTGAGTCAAGCCAA** | **qRT-PCR** |
